# Unambiguous identification of asymmetric and symmetric synapses using volume electron microscopy

**DOI:** 10.3389/fnana.2024.1348032

**Published:** 2024-04-05

**Authors:** Nicolás Cano-Astorga, Sergio Plaza-Alonso, Marta Turegano-Lopez, José Rodrigo-Rodríguez, Angel Merchan-Perez, Javier DeFelipe

**Affiliations:** ^1^Laboratorio Cajal de Circuitos Corticales, Centro de Tecnología Biomédica, Universidad Politécnica de Madrid, Madrid, Spain; ^2^Instituto Cajal, Consejo Superior de Investigaciones Científicas (CSIC), Madrid, Spain; ^3^PhD Program in Neuroscience, Autonoma de Madrid University-Cajal Institute, Madrid, Spain; ^4^Centro de Investigación Biomédica en Red de Enfermedades Neurodegenerativas (CIBERNED), Instituto de Salud Carlos III, Madrid, Spain; ^5^Departamento de Arquitectura y Tecnología de Sistemas Informáticos, Universidad Politécnica de Madrid, Madrid, Spain

**Keywords:** cerebral cortex, 3D-electron microscopy, FIB-SEM, excitatory and inhibitory synapses, potassium ferrocyanide, ultrastructure, VGAT

## Abstract

The brain contains thousands of millions of synapses, exhibiting diverse structural, molecular, and functional characteristics. However, synapses can be classified into two primary morphological types: Gray’s type I and type II, corresponding to Colonnier’s asymmetric (AS) and symmetric (SS) synapses, respectively. AS and SS have a thick and thin postsynaptic density, respectively. In the cerebral cortex, since most AS are excitatory (glutamatergic), and SS are inhibitory (GABAergic), determining the distribution, size, density, and proportion of the two major cortical types of synapses is critical, not only to better understand synaptic organization in terms of connectivity, but also from a functional perspective. However, several technical challenges complicate the study of synapses. Potassium ferrocyanide has been utilized in recent volume electron microscope studies to enhance electron density in cellular membranes. However, identifying synaptic junctions, especially SS, becomes more challenging as the postsynaptic densities become thinner with increasing concentrations of potassium ferrocyanide. Here we describe a protocol employing Focused Ion Beam Milling and Scanning Electron Microscopy for studying brain tissue. The focus is on the unequivocal identification of AS and SS types. To validate SS observed using this protocol as GABAergic, experiments with immunocytochemistry for the vesicular GABA transporter were conducted on fixed mouse brain tissue sections. This material was processed with different concentrations of potassium ferrocyanide, aiming to determine its optimal concentration. We demonstrate that using a low concentration of potassium ferrocyanide (0.1%) improves membrane visualization while allowing unequivocal identification of synapses as AS or SS.

## 1 Introduction

Cortical synapses exhibit a wide range of structural, molecular, and functional characteristics. Nevertheless, they can be classified into two primary morphological types: Gray’s type I and type II (Gray, 1959), corresponding to Colonnier’s asymmetric (AS) and symmetric (SS) synapses, respectively (Colonnier, 1968). The most noticeable distinction lies in the postsynaptic density: AS have a thick postsynaptic density, while SS have a thin postsynaptic density. In general, regarding all synapses in the neuropil of the cerebral cortex, AS outnumber SS approximately 95:5 (see Alonso-Nanclares et al., 2023, and Cano-Astorga et al., 2023, and the references therein). Importantly, it has been demonstrated that, in the cerebral cortex, most AS are excitatory (glutamatergic), while SS are inhibitory (GABAergic) (Colonnier, 1968; Gray, 1969; Peters and Kaiserman-Abramof, 1969; Houser et al., 1984; Peters and Palay, 1996; Ascoli et al., 2008). Additionally, the size of synapses is correlated with various functional aspects, including the probability of neurotransmitter release, synaptic strength, efficacy, the number of postsynaptic receptors, and plasticity (e.g., see Santuy et al., 2018a, and Chindemi et al., 2022, and references therein). Therefore, determining the distribution, size, density and proportion of the two major cortical types of synapses is of vital importance, not only for a better understanding of synaptic organization in terms of connectivity but also from a functional perspective. However, there are several technical challenges that complicate the study of synapses when conventional electron microscopy methods —which provide only 2D image data— are used. For instance, in individual sections, the synaptic cleft and the densities in the pre-and postsynaptic membranes appear blurred in a significant proportion of synaptic junctions, ranging from 40 to 60%. As previously discussed in DeFelipe et al. (1999), this is because in these single section cases, the planes of section are not passing at right angles to the synaptic junctions, with the extreme case being the *en face* view (plane of section parallel to the plane of the synaptic junction). Furthermore, SS are the most challenging type to identify, as AS may resemble SS in certain planes of section. Nevertheless, through the examination of the same synapse using serial sections, numerous studies have successfully distinguished these junctional complexes as either AS or SS types (e.g., Merchán-Pérez et al., 2009, and references contained therein). The challenge lies in obtaining long series of thin sections to distinguish between AS and SS and accurately estimate their density. As a result, various stereological methods have been developed over the years to estimate synapse density and the proportion of AS and SS (DeFelipe et al., 1999). Fortunately, the recent introduction of automated volume electron microscopy methods has proven to be a valuable and efficient approach for identifying synapses in three dimensions, becoming the gold standard technique for this purpose for this task (Merchán-Pérez et al., 2009).

Another crucial factor to consider is the proper preservation of synaptic membranes, a prerequisite for effectively distinguishing between AS and SS based on their morphological differences. Various protocols for brain perfusion have been employed over the years to achieve this preservation. Importantly, the primary fixative used does not override the main feature utilized for distinguishing between AS and SS, which is the thickness of electron-dense labeling in the postsynaptic density (PSD). Various heavy metals have long been employed for post-fixation and positive staining of biological materials in electron microscopy (Hall et al., 1945; Gibbons and Bradfield, 1956; Watson, 1958; Reynolds, 1963). Among them, osmium tetroxide (OsO_4_) plays a key role in the fixation of lipids, proteins, lipoproteins, nucleic acids and carbohydrates, and acts as a bridge to allow the precipitation of contrasting agents such as additional osmium, uranyl or lead (Hayat and Giaquinta, 1970). Later, the introduction of potassium ferrocyanide (potassium hexacyanoferrate (II); K4[Fe(CN)6]; Karnovsky, 1971; White et al., 1979; McDonald, 1984) or potassium ferricyanide (potassium ferricyanide (III); K3[Fe(CN)6]; Rivlin and Raymond, 1987) in combination with osmium tetroxide was used to enhance the visualization of cellular membranes, as well as certain aspects of cell morphology. Furthermore, it has been reported that the use of either reagent in combination with osmium tetroxide work equally well (Rivlin and Raymond, 1987), increasing the quality of electron microscopy images. However, it should be noted that the use of potassium ferrocyanide or ferricyanide is not necessary for the morphological identification of synapses (see, for example, Peters et al., 1991).

Here, we describe a protocol for the preparation of brain tissue fixed with paraformaldehyde to be studied with Focused Ion Beam Milling and Scanning Electron Microscopy (FIB-SEM). This technology was chosen because it enables automated serial sectioning of large volumes of tissue, without any mechanical interaction with the sample (e.g., see Knott et al., 2008; Merchán-Pérez et al., 2009; Titze and Genoud, 2016; Kubota et al., 2018; Rollenhagen et al., 2020). In this study, we describe in detail the brain tissue preparation for electron microscopy, the FIB-SEM serial imaging procedure, and the identification and segmentation of synapses. We focus on the unambiguous identification of AS and SS, based on morphological criteria. In a prior study, we conducted pre-embedding immunocytochemical labeling of the vesicular GABA transporter (VGAT) in fixed sections of mouse brain tissue. Subsequently, we used FIB-SEM to image cortical regions with VGAT-positive puncta, identifying synapses based on VGAT-positive boutons and unlabeled terminals. This material was prepared without potassium ferrocyanide, and the AS and SS were clearly identified and distinguished from one another (Turégano-López et al., 2021). However, volume electron microscopy studies have commonly employed potassium ferrocyanide (Harris and Stevens, 1988, 1989; Harris et al., 1992, 2015; Medalla et al., 2007; Hua et al., 2015) or potassium ferricyanide (Tapia et al., 2012). These compounds facilitate the reconstruction of cellular processes and the automatic segmentation of electron microscope images. To confirm that the SS observed with our FIB-SEM protocol (which includes potassium ferrocyanide) were indeed GABAergic, we conducted a series of experiments with different concentrations of potassium ferrocyanide, aiming to determine the optimal concentration.

## 2 Materials and methods

### 2.1 Equipment

The main equipment used to set up the technique was as follows: Vibratome (Leica VT 1200S); Variable Wattage Microwave (PELCO BioWave Pro 36,500–230); Ultramicrotome (Leica EM UC6); Diamond Knive (Diatome Histo #5961); Sputter Coater (Quorum Emitech SC7620); and Focused Ion Beam – Scanning Electron Microscope (FIB-SEM; Zeiss, CrossBeam 540).

### 2.2 Solutions

0.1 M phosphate buffer solution (PB): the solution contains 2.65 g of sodium di-hydrogen phosphate 1-hydrate (PanReac #131965) and 14 g of di-potassium hydrogen phosphate (PanReac #121512) in 1 L of distilled H2O; pH 7.4.

Perfusion fixation solution: 4% paraformaldehyde (PFA; Electron Microscopy Sciences #15714-S) in PB. The solution must be prepared just before use in a fume hood.

First postfixation solution: 4% PFA in PB. Prepare just before use in a fume hood.

Sectioning solution: 10% sucrose (PanReac #57501) in PB.

Cryoprotection solution: 30% sucrose in PB.

Preincubation solution: 3% bovine serum albumin (BSA; Sigma #A4503-50G) in PB.

Primary antibody solution: rabbit anti-Vesicular GABA Transporter Antibody (VGAT; Synaptic Systems #131003; 1:2000) and 3% BSA in PB.

Positive control of primary antibody solution: rabbit anti-parvoalbumin (PV; ABCAM #AB11427; 1:1000) and 3% BSA in PB.

Secondary antibody solution: biotinylated goat anti-rabbit IgG antibody (Vector Laboratories #BA-1000; 1:200) and 3% BSA in PB.

Avidin-Biotin Complex (ABC)-based detection method: the solution contains 0.008% reagent A (Avidin; ABC Elite) and 0.008% reagent B (biotinylated HRP, ABC Elite) from the ABC kit (Vector Laboratories #PK-6100) in PB. Prepare 30 min before use.

Preincubation solution of 3,3′-Diaminobenzidine (DAB): 0.05% DAB (Sigma #D5905) in PB. Prepare immediately before use in a fume hood and protect from light. Filter with a syringe filter (Acrodisc 0.2 μm, #4612) before use.

Incubation solution of DAB: immediately before use, add 0.01% hydrogen peroxide (H2O2; Merck # 1.07209.1000) to the DAB solution described above and mix well.

Second postfixation solution: freshly prepared 4% PFA, 0.2% glutaraldehyde (GA; TAAB #G002), and 0.003% calcium chloride (CaCl2; Sigma #C-2661) in 0.1 M cacodylate buffer (Sigma #C0250). Prepare in a fume hood.

Microwave postfixation solution: freshly prepared 2% PFA, 2.5% GA, and 0.003% CaCl2 in 0.1 M cacodylate buffer. Prepare in a fume hood.

First osmium solution — prepared with or without potassium ferrocyanide: 1% OsO4 (Sigma #O5500), 0, 0.1% or 1% potassium ferrocyanide (Probus #23345) and 0.003% CaCl2 in 0.1 M cacodylate buffer. Always handle osmium and potassium ferrocyanide in a fume hood, with protective glasses and double gloves.

Second osmium solution: 1% OsO4 and 0.003% CaCl2 in 0.1 M cacodylate buffer. Always handle osmium in a fume hood, with protective glasses and double gloves.

Uranyl acetate solution for en bloc staining: the solution contains 1% uranyl acetate (Electron Microscopy Sciences #22400) in 50, 70, 90 and 100% ethanol. Filter with a syringe filter (Acrodisc 0.2 μm, #4612).

Silver paint (Electron Microscopy Sciences, #12630).

### 2.3 Animals, perfusion fixation, and vibratome sectioning

We used four adult female mice (C57BL/6, 8 weeks old) for the technique outlined in this study. Two of these mice were utilized to assess various concentrations of potassium ferrocyanide, with one mouse assigned to each condition. The remaining two mice were dedicated to VGAT validation — one with potassium ferrocyanide (0.1%) and the other without potassium ferrocyanide. All animal handling procedures were conducted in accordance with the guidelines for animal research outlined in the European Community Directive 2010/63/EU, and all procedures were approved by the Local Ethics Committee of the Spanish National Research Council (CSIC).

To begin the procedure, anesthetize the animals with an intraperitoneal injection of pentobarbital (40 mg/kg) and then intracardially perfuse with 100 mL of freshly prepared fixation solution (4% PFA in 0.1 M PB). Postfix the brains for 6 to 16 h (overnight) in the postfixation solution (4% PFA in 0.1 M PB). Then, cut the brains into sections (150 μm thick) using a vibratome and collect them in a sectioning solution (sucrose 10%, in 0.1 M PB) in 24-well flat-bottom plates.

### 2.4 Postfixation and osmication

This part of the procedure was carried out as follows: (1). Postfix the sections for 48 h at 4°C in the second postfixation solution (4% PFA, 0.2% GA and 0.003% CaCl2 in 0.1 M cacodylate buffer). (2) Wash the sections (three times, 10 min each) in 0.1 M cacodylate buffer. (3) Perform microwave postfixation by placing the sections in the microwave postfixation solution (2% PFA, 2.5% GA, and 0.003% CaCl2 in 0.1 M cacodylate buffer) for 1 min at 50°C using the variable wattage microwave at 150 W power. Carefully add the fixative using a plastic Pasteur pipette without agitating the sections to prevent curling or folding. This step should be conducted in a fume hood. (4) Wash the sections three times in 0.1 M cacodylate buffer, 10 min each wash. (5) Osmicate the sections for 1 h in the first osmium solution in a fume hood. Slowly add and remove the osmium solution using a plastic Pasteur pipette to avoid folding or breaking the sections. Note that during osmication, sections become brittle and should be handled with care, using a small spatula or weighing spoon. (6) Wash the sections three times in 0.1 M cacodylate buffer, 10 min each wash. (7) Osmicate the sections again for 1 h with the second osmium solution in a fume hood.

### 2.5 Dehydration, en bloc staining and embedding

Using a variable wattage microwave at 50°C, 250 W power, dehydrate the sections in a series of uranyl acetate solutions, starting with 50% ethanol and continue with a solution of 1% uranyl acetate in increasing ethanol concentrations (50–70%-90–100%), finishing with absolute ethanol and clear three times in acetone (40 s each step). Embed the sections in Araldite, with a variable wattage microwave (under vacuum conditions at 70°C, 350 W power, 3 min each step), as follows: solution of 1 part Araldite, 1 part acetone — followed by a solution of 4 parts Araldite and 1 part acetone and finishing with a 3-step embedding with pure Araldite. Store the sections embedded in pure Araldite at 4°C for 8–16 h (overnight).

### 2.6 Flat-embedding and re-sectioning

Temper the sections for 30 min at room temperature. Change the Araldite to a freshly prepared mixture and leave the sections to rest for three to 4 h. Flat-embed each section by placing them between two silicone coater slides covered with a transparent film for 48 h at 60°C. To ensure that the flat-embedding is homogenous, distribute small weights over the slide.

Once the resin has cured, the flat-embedded sections must be examined and photographed under an optical microscope to select the region of interest. Then, trim and glue the region of interest (in this case: the primary somatosensory cortex) with cyanoacrylate onto a blank Araldite block. Use a microtome and a diamond knife to obtain serial semithin sections, until reaching the tissue. Photograph the surface of the block to establish landmarks (such as blood vessels or other morphological features), which will later be used to precisely locate the area to be imaged with the FIB-SEM.

### 2.7 Focused ion beam milling and scanning electron microscopy imaging

Once the region of interest has been selected in the Araldite block, mount it on an SEM specimen stub with a conductive carbon sticker (Electron Microscopy Sciences, #77825–09). To prevent charge build-up, the block must be covered with silver paint, except for the top surface. It is important not to cover or spill silver droplets on the upper surface of the block where the specimen is located. Conversely, the base of the block must be carefully painted to ensure electrical continuity between the Araldite block and the specimen stub. Allow the paint to dry for at least 24 h in a vacuum desiccator. Charge dissipation from the upper surface of the block is achieved by gold–palladium sputter-coating for 60 s. Carbon, gold alone, or other metals are also suitable for sputter coating, but care must be taken not to cover the specimen with a layer that is too thick as this might obscure surface details.

The surface of the block is then photographed with the SEM using the secondary electron detector. The landmarks in the section that were previously identified with the optical microscope (mainly small blood vessels) are also visible with the SEM, so the region of interest can be precisely located. A viewing trench is then excavated with the FIB using a 7 nA milling current, to provide visual access to the region that we plan to image. The front face of this trench must be located close enough to the target to allow its identification. The ion beam and the electron beam can be used simultaneously, so it is possible to monitor the progression of the trench as it is being excavated. As soon as we have identified our target, milling of the viewing trench is stopped. We then use a smaller FIB current (700 pA) to progressively mill the front face of the trench in steps of 20 nm. During each milling step, we remove 20 nm of material with the FIB, and then use the SEM to take a microphotograph of the freshly milled surface. In our equipment, the angle between the SEM and the FIB is 54°, so the angle of incidence of the SEM on the surface to be imaged is 36°, rather than perpendicular. The resulting perspective deformation is automatically corrected by the microscope software during acquisition (SmartSEM 6.02; Carl Zeiss Microscopy Ltd.), so no distortion is present in the final images.

Since the milling/imaging cycle can be fully automated, serial images of the target are obtained. We routinely use a milling step of 20 nm (equivalent to section thickness) and a resolution in the X-Y plane of 5 nm/pixel, so the actual voxel size is 5 nm × 5 nm × 20 nm (Merchán-Pérez et al., 2009). Other resolutions and milling steps can also be used, depending on the particular imaging needs, and the length of the series of sections can be selected according to the researcher’s needs.

Some drift may occur during the acquisition of the FIB-SEM image series. In this case, further alignment (registration) is required. For the registration process, programs such as FIJI, a distribution of ImageJ with preinstalled plugins for microscopy (Schindelin et al., 2012), can be used. We recommend setting a “rigid” registration protocol, with translation only allowed for the alignment, as this avoids deformation and rotation of individual images. The aligned stack of images is then visualized in EspINA software (Morales et al., 2011), which allows synaptic identification and segmentation through the original plane of section or the other two orthogonal planes (EspINA Interactive Neuron Analyzer, 2.9.12; https://cajalbbp.csic.es/espina-2).

A synapse is recognized according to well-established criteria (e.g., see Colonnier, 1981; Peters et al., 1991; Peters and Palay, 1996). The identification process involves confirming the presence of specific elements, including densities on the cytoplasmic faces in the pre-and postsynaptic membranes; synaptic vesicles in the presynaptic axon terminal adjacent to the presynaptic density; and a synaptic cleft. Generally, three types of structural units are employed for synapse identification (see Mayhew, 1996, for a review): terminal boutons, total apposition zones, and synaptic membrane densities. In this study, we primarily use synaptic membrane densities for synapse counting, especially when accompanied by synaptic vesicles near the presynaptic density, irrespective of the angle of section through which the synaptic junctions are viewed (i.e., whether a synaptic cleft is evident or not). Moreover, the identification of synapses relies on examining all serial sections where each individual synapse is visible. Additionally, utilizing EspINA software, the 3D course of the axons can be followed within stacks of sections to confirm the nature (AS or SS) of the synapses established in all their synaptic contacts.

### 2.8 Pre-embedding immunohistochemistry

We conducted pre-embedding immunocytochemical labeling of VGAT in fixed brain tissue sections and subsequently processed the tissue for FIB/SEM, as described above, with the following modifications to investigate the synaptic contacts established by VGAT-positive boutons. After vibratome sectioning, 150 μm-thick sections are cryoprotected using sucrose 30% in 0.1 M PB overnight. Permeabilize sections using liquid nitrogen. Place two to three brain sections in 5 mL Eppendorf tubes. Then, remove the remaining sucrose solution and ensure that the sections are distributed along the Eppendorf tube surface, clearly separated and stretched. Immerse the Eppendorf tubes in the liquid nitrogen solution for 2 or 3 s. Immediately after the cryopermeabilization, store the Eppendorf tubes at 4°C. The sections must have a white, opaque appearance. Once the sections return to their usual transparent appearance, slowly add 0.1 M PB (4°C) to the Eppendorf tube and store again at 4°C.

Carefully place the permeabilized section in 24-well flat-bottom plates filled with 0.1 M PB. Wash the sections (three times, 10 min each) in 0.1 M PB, under agitation. Then, pre-incubate the sections with the preincubation solution (3% BSA, in 0.1 M PB) for 2 h under agitation at room temperature. Incubate with the primary antibody incubation solution (rabbit anti-Vesicular GABA Transporter Antibody, in 3% BSA - 500 μL/section) for 48 h under agitation at 4°C. A positive control is recommended to exclude any possible miscoupling during the antibody reaction.

Allow the sections to temper for 10–15 min at room temperature. Wash the sections (three times, 10 min each) with 3% BSA, in 0.1 M PB. Incubate the sections with the secondary biotinylated antibody solution (biotinylated goat anti-rabbit IgG antibody, in 3% BSA), for 2 h under agitation at room temperature. Wash the sections (three times, 10 min each) with 0.1 M PB. Incubate the sections with the Avidin-Biotin Complex (ABC)-based detection kit to amplify the secondary antibody signal, for 1 h under agitation at room temperature. Wash three times in 0.1 M PB at room temperature, 10 min each time. In a fume hood, pre-incubate the sections in DAB solution without H2O2, protected from light. Next, incubate in DAB solution with H2O2 for 1 min. The sections will change to a brown, whiskey-like appearance, so visually monitor the color of the sections until the precipitate has reached the desired intensity. This can also be checked using an optical microscope. Stop the reaction by washing the sections three times (10 min each) in 0.1 M PB. Once the immunostaining is checked, follow the processing procedure for electron microscopy: postfixation and osmication are performed as described above, but adding 7% glucose (Merck #1.08337.0250 in the first and second osmium solutions to avoid excessive darkening of the sections). The first osmium solution contains 0.1% potassium ferrocyanide. Dehydration, en bloc staining and embedding are performed as described above.

## 3 Results

### 3.1 Identification of As and SS synapses using FIB/SEM on unlabeled brain tissue

**Figure 1:**
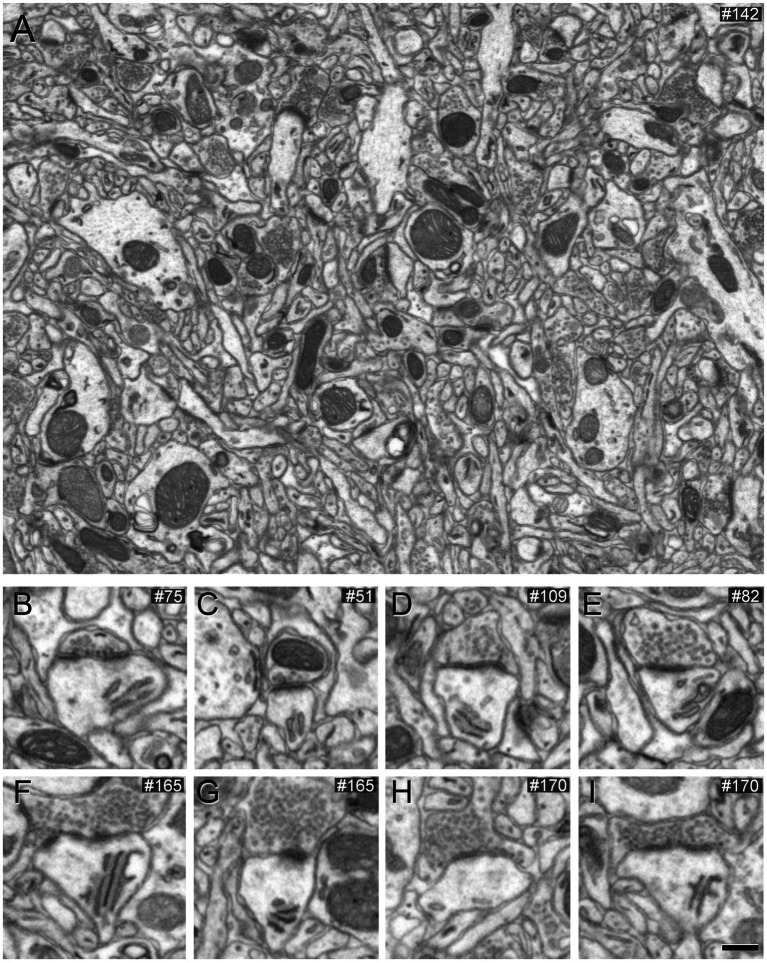
Images obtained by FIB/SEM showing the neuropil of the somatosensory cortex of mice. The sample was treated with 1% potassium ferrocyanide and not permeabilized with liquid nitrogen. **(A)** Low-magnification FIB/SEM image from a stack to illustrate the good quality of the EM image. **(B–I)** Various examples of synapses on different dendritic spines, which typically establish AS. However, in this material, AS are challenging to identify because the postsynaptic densities are relatively thin. Scale bar (in **I**) indicates 468 nm for **(A)**, and 315 nm for **(B–I)**.

**Figure 2:**
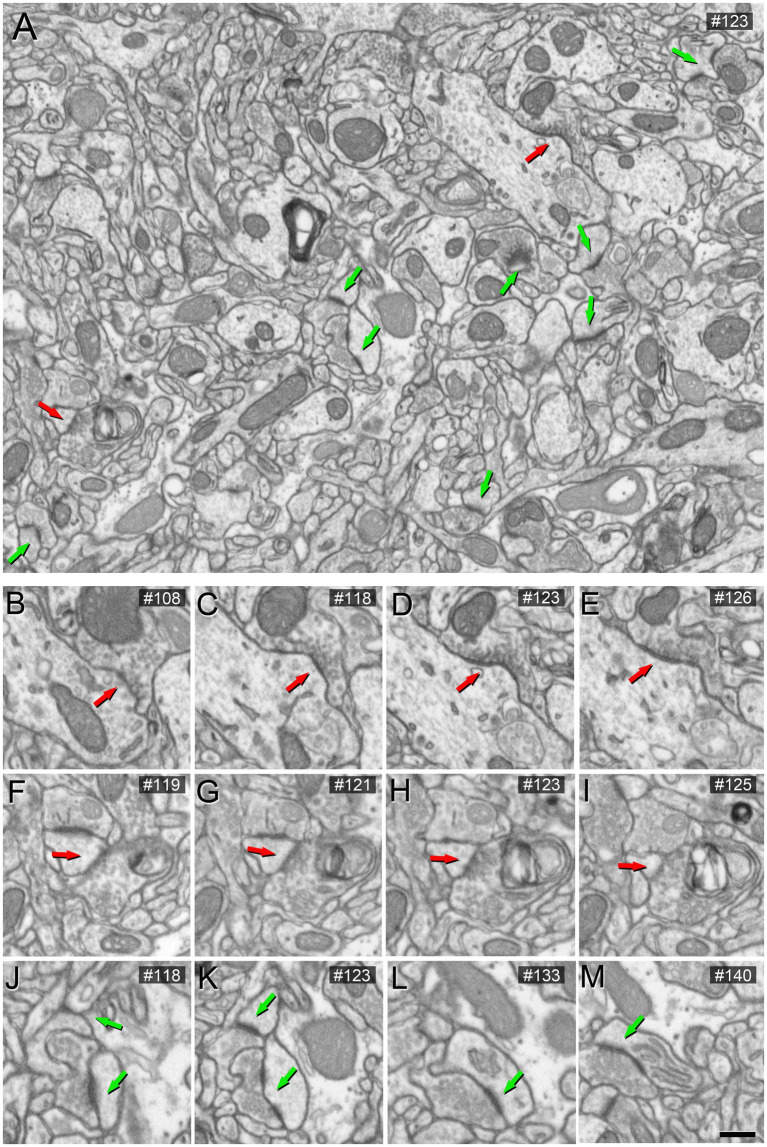
Images obtained by FIB/SEM showing the neuropil of the somatosensory cortex of mice. The sample was treated with 0.1% potassium ferrocyanide and not permeabilized with liquid nitrogen. In **(A)**, an example of a low-magnification FIB/SEM image from a stack highlights AS and SS synapses with green and red arrowheads, respectively. **(B–I)** Various serial sections at higher magnification of the same SS (red arrow). **(J–M)** Various serial sections of the same AS (green arrow) from the image stack. The section number is indicated in the top right-hand corner of each image. Scale bar (in **M**) indicates 468 nm for **(A)**, and 315 nm for **(B–M)**.

To assess the impact of different concentrations of potassium ferrocyanide on the appearance of synaptic junctions, we conducted the study on layers II and III. A detailed examination of 201 slices (covering a total volume of 361.68 μm^3^) from an image stack treated with a concentration of 1% potassium ferrocyanide revealed excellent EM image quality, primarily due to clearly thickened membranes. However, identifying SS proved challenging as their thin PSD exhibited a thickness similar to the surrounding non-synaptic membranes. In the case of AS, they remained visible, but their PSD appeared thinner compared to the thicker surrounding membranes (Figure 1; Supplementary Figures S2.1A, S2.2A, S2.3A, S2.4A). Consequently, we explored lower concentrations of potassium ferrocyanide. We examined 299 slices (covering a total volume of 538.02 μm^3^) from an image stack treated with a concentration of 0.1% potassium ferrocyanide. As illustrated in Figure 2 (see also Supplementary Figures S2.1B, S2.2B, S2.3B, S2.4B), at a concentration of 0.1%, the quality of the EM images remained excellent, and AS and SS could be clearly distinguished through serial sections. In this image stack, we unambiguously identified 88 SS and 836 AS.

### 3.2 VGAT pre-embedding immunocytochemistry

Next, brain sections that were labeled for VGAT and processed for EM using 0.1% potassium ferrocyanide were imaged using FIB-SEM to examine the morphology of the synaptic junctions established by VGAT-positive boutons — and to compare with the morphology of the synapses of unlabeled axon terminals. As has been shown previously (e.g., Takayama and Inoue, 2010), VGAT immunoreactivity was distributed across all layers of the mouse primary somatosensory cortex, where numerous stained puncta were scattered in the neuropil (Supplementary Figure S1A). In layers II to VI, especially in layer V, positive puncta were distributed both in the neuropil and around unlabeled somata and their proximal processes (Supplementary Figure S1B).

**Figure 3:**
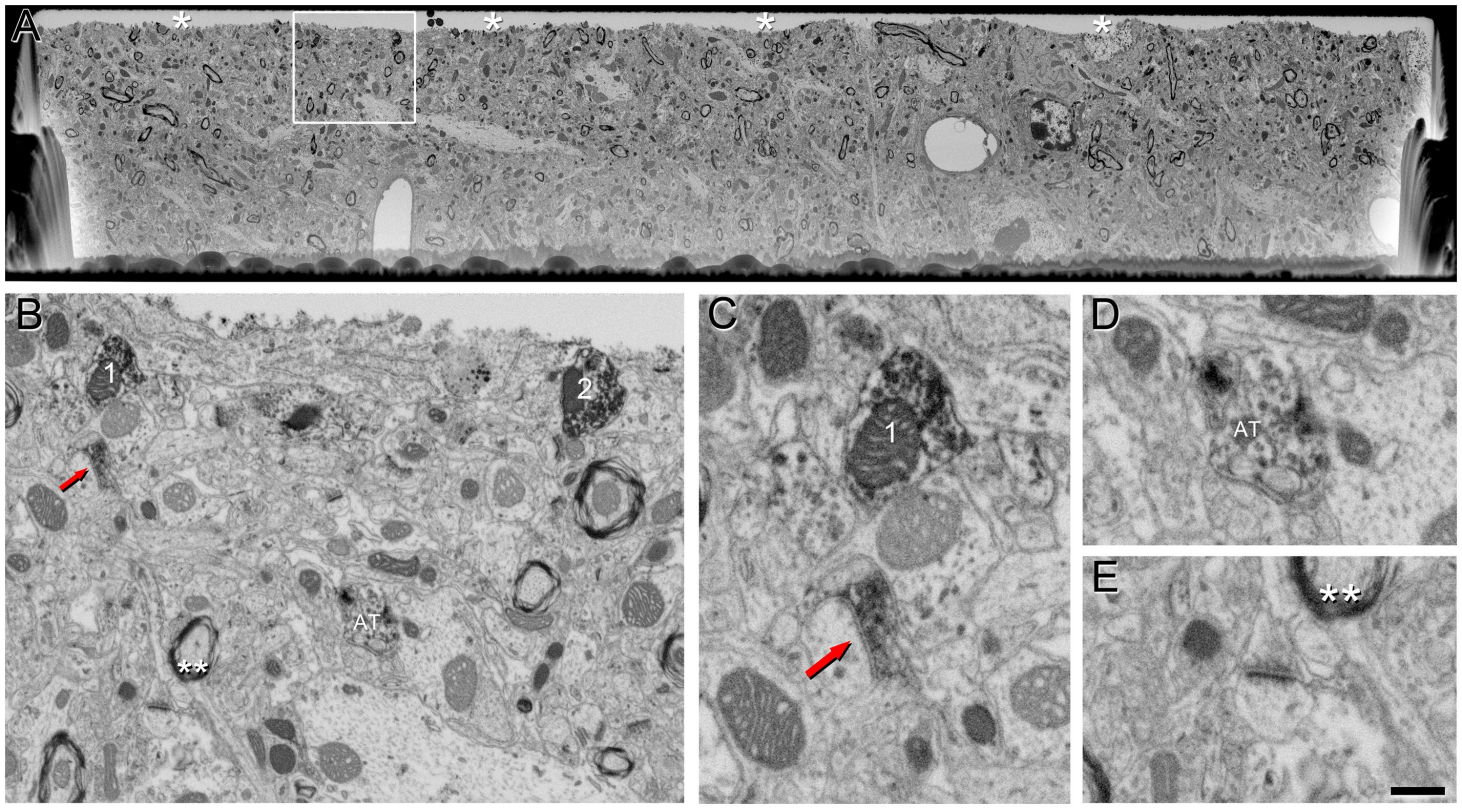
VGAT-positive axon terminals in a single SEM image from tissue treated with 0.1% potassium ferrocyanide and permeabilized with liquid nitrogen. **(A)** A viewer trench was excavated using FIB milling on the surface of a brain section. The asterisks indicate the interface between the embedding medium (Araldite) and the brain tissue. **(B)** Higher magnification of the boxed area in **A**, showing the neuropil. Axon terminals 1 and 2 establish synapses with a cell body that becomes more apparent through the serial sections (see Figure 5). The red arrow indicates a VGAT-positive terminal forming an SS (magnified in **C**), AT indicates another VGAT-positive terminal (magnified in **D**), and double asterisks indicate neuropil magnified in **E**. **(C)** Higher magnification of the VGAT-positive terminal forming an SS (red arrow), and the VGAT-positive terminal (1) forming an SS with the cell somata in further serial sections shown in **B**. **(D)** Example of a VGAT-positive terminal in which the intensity of the staining decreases as the distance to the surface of the section increases. **(E)** Example of a VGAT-negative terminal forming an AS. Scale bar (in **E**) indicates 5 μm for **(A)**, 800 nm for **(B)** and 370 nm for **(C–E)**.

We conducted FIB/SEM analyses in the region that exhibited strong VGAT-immunoreactivity using correlative light-electron microscopy methods. A viewer trench was excavated with the FIB (Supplementary Figure S1C) to image the tissue within the penetration zone of the immunostaining. In this zone, VGAT-positive axon terminals filled with dark immunostained vesicles can be visualized (Figure 3). The intensity of the staining in these terminals decreases as the distance to the surface of the section increases (Figure 3B).

**Figure 4:**
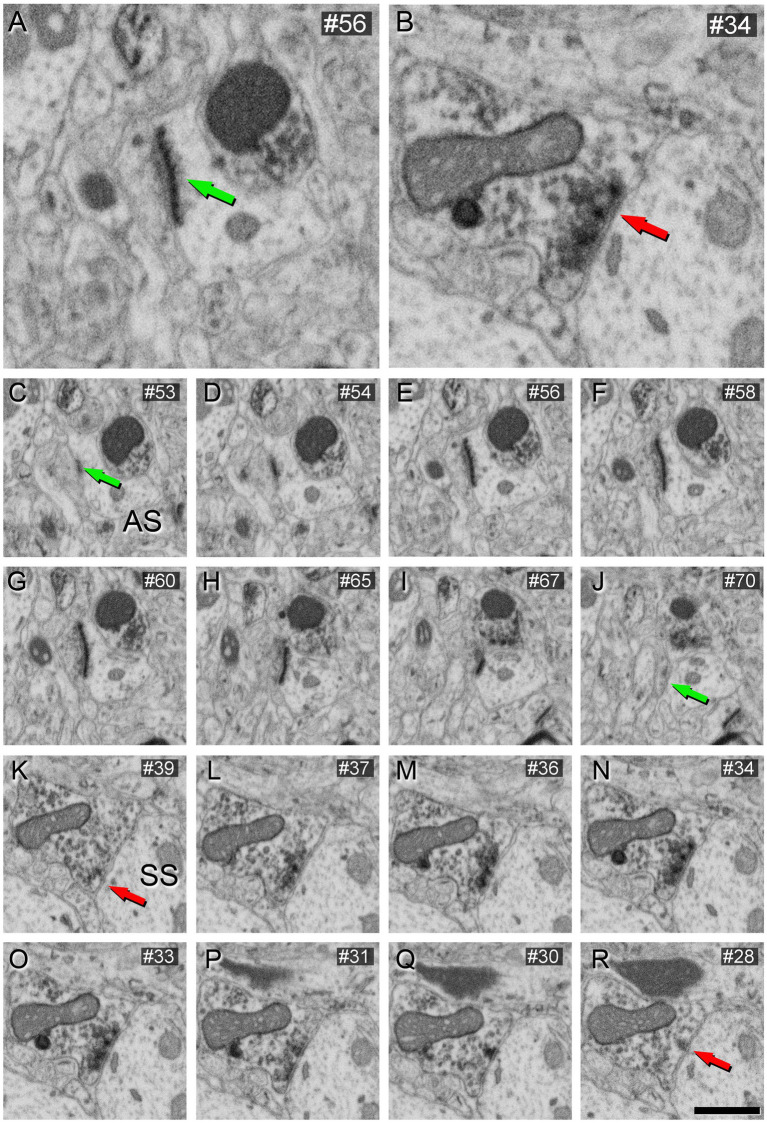
AS **(A)** and SS **(B)** identification from FIB/SEM images in VGAT-immunostained tissue permeabilized with liquid nitrogen and treated with 0.1% potassium ferrocyanide. Sequence of FIB-SEM serial images of an AS **(C–J)** and an SS **(K–R)**. Numbers on the top right of each panel indicate the number of each section from the stack of FIB/SEM images. Synapse classification was performed based on the thickness of the PSD and the VGAT-positive labeling of the presynaptic terminal through the examination of full sequences of serial images. Green arrows indicate the beginning **(C)** and the end **(J)** of the AS. Red arrows indicate the beginning **(K)** and the end **(R)** of the SS. Note the VGAT-positive presynaptic staining on the SS. Scale bar (in **R**) indicates 250 nm for **(A,B)**, and 500 nm for **(C–R)**.

These VGAT-positive terminals established SS, while VGAT-negative axon terminals established AS (Figure 4).

**Figure 5:**
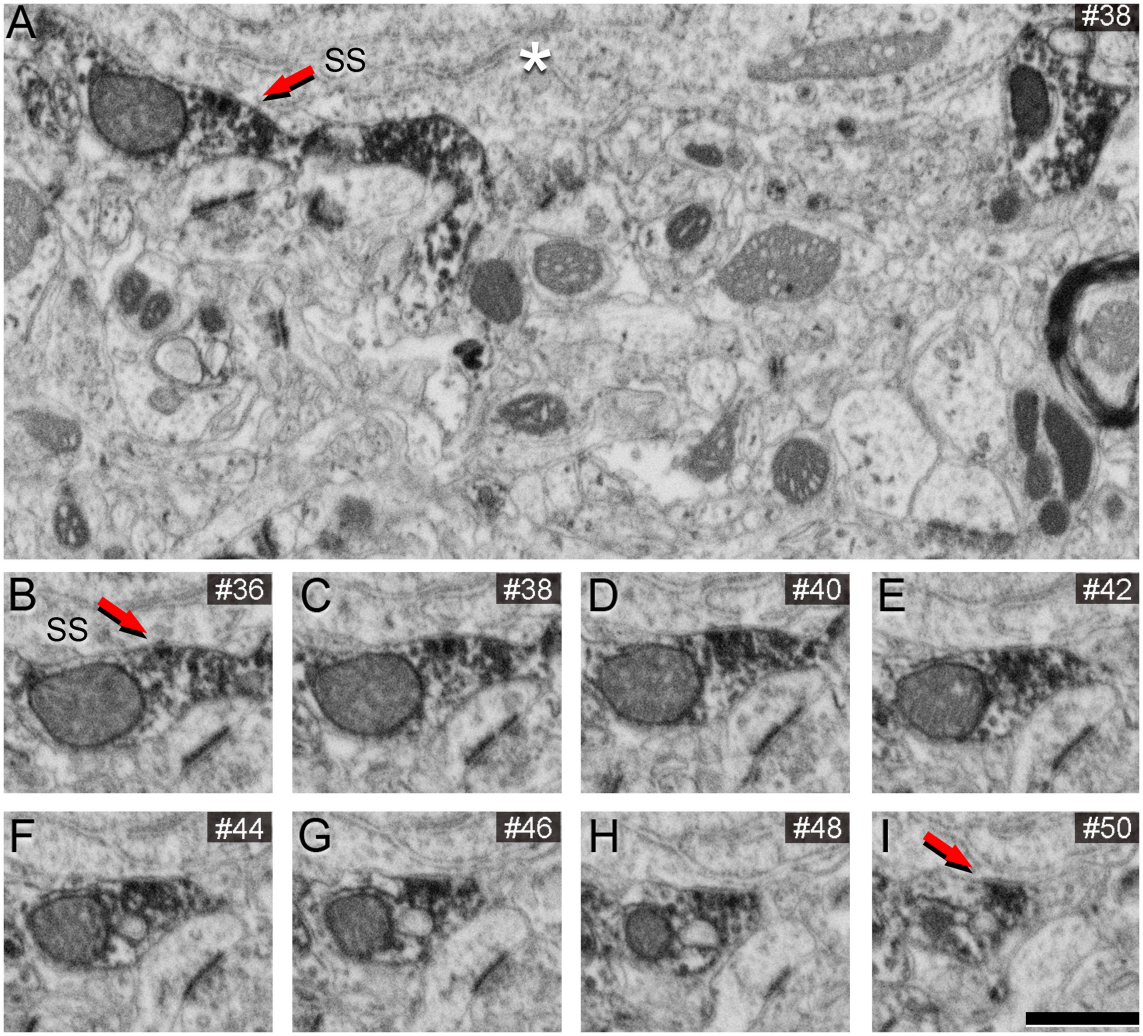
Identification of SS on neuronal soma from FIB/SEM images in VGAT-immunostained tissue permeabilized with liquid nitrogen and treated with 0.1% potassium ferrocyanide. White asterisk indicates the neuronal soma. **(B–I)** sequence of FIB-SEM serial images of an SS established on the neuronal soma. Numbers on the top right of each panel indicate the number of each section from the stack of FIB/SEM images. Red arrows indicate the beginning **(B)** and the end **(I)** of the SS. Scale bar (in **I**) indicates 520 nm for **(A)**, and 500 nm for **(B–I)**.

Further verification of the morphology of synaptic contacts made by VGAT-positive boutons in the neuropil was obtained by examining the perisomatic innervation of pyramidal cells by VGAT-immunoreactive axon terminals, where it is known that only SS are established (DeFelipe and Fariñas, 1992). As shown in Figure 5, these perisomatic axon terminals clearly established SS, as expected, and were identical to those found in the neuropil (Figure 4).

**Figure 6:**
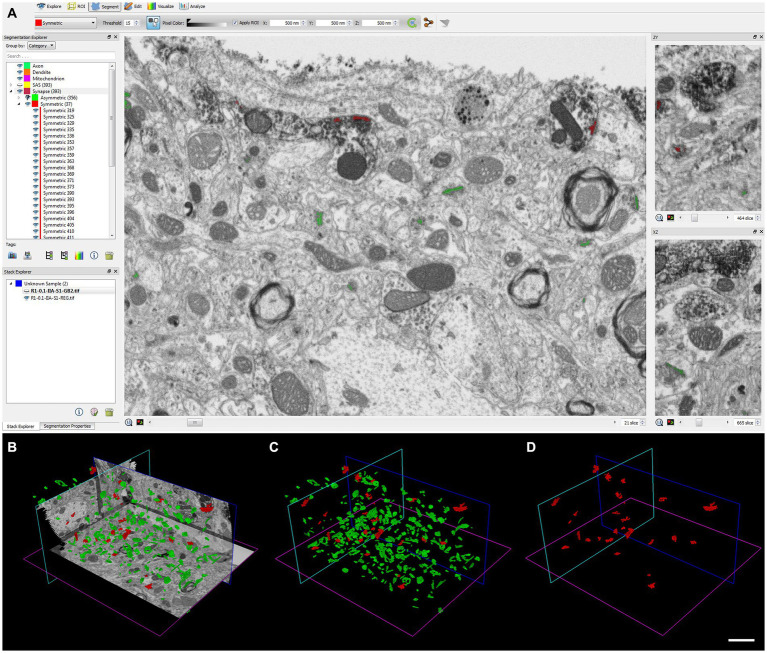
Identification and segmentation of synapses. **(A–D)** Screenshots of the EspINA software user interface. **(A)** In the main window, the sections are viewed through the xy plane (as obtained by FIB/SEM microscopy). The other two orthogonal planes, yz and xz, are also shown in adjacent windows (on the right). **(B)** 3D view showing the three orthogonal planes and the 3D reconstruction of AS (green) and SS (red) segmented synaptic junctions. **(C)** 3D reconstructed synaptic junctions of both AS and SS, displayed using the same colors as in **B**. **(D)** 3D reconstructed synaptic junctions of SS. Scale bar (in **D**) indicates 2 μm for **(B–D)**.

We analyzed 266 serial images in the neuropil within the penetration zone of immunostaining, corresponding to 4,184 μm^3^, and identified 265 AS and 23 SS. All SS (8% of total synapses) were formed by VGAT-positive terminals, while all AS (92%) were established by VGAT-negative terminals (Figure 6).

**Figure 7:**
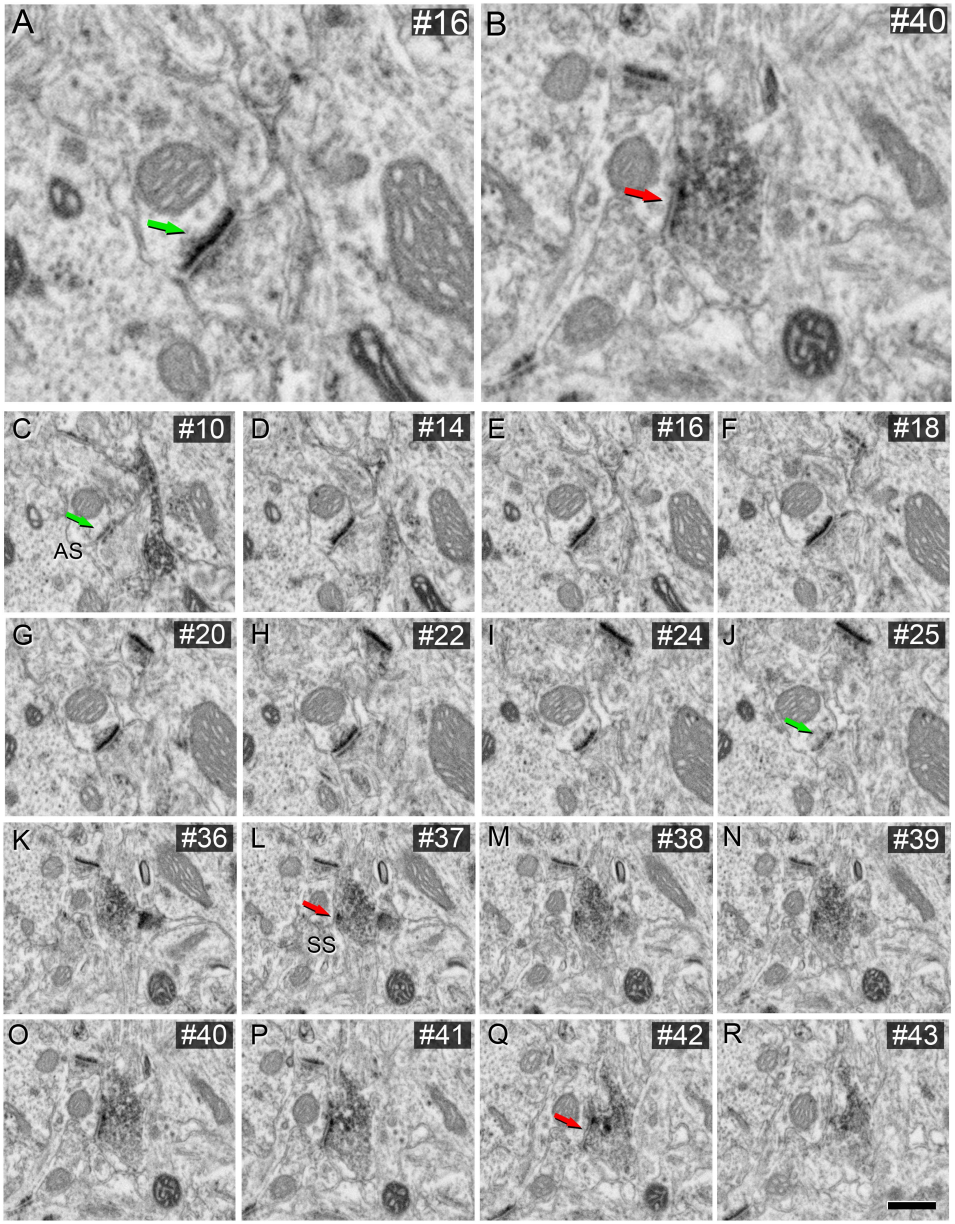
AS **(A)** and SS **(B)** identification from FIB/SEM images in VGAT-immunostained tissue permeabilized with liquid nitrogen and not treated with potassium ferrocyanide. FIB-SEM serial images of an AS **(C–J)** and an SS **(K–R)** are shown. Numbers on the top right of each panel indicate the number of each section from the stack of FIB/SEM images. Synapse classification was performed based on the thickness of the PSD and the VGAT-positive labeling of the presynaptic terminal through the examination of full sequences of serial images. Green arrows indicate the beginning **(C)** and the end **(J)** of the AS. Red arrows indicate the beginning **(K)** and the end **(R)** of the SS. Note the VGAT-positive presynaptic staining on the SS. Scale bar (in **R**) indicates 230 nm for **(A)**, 270 nm for **(B)**, 253 nm for **(C–J)**, and 540 nm for **(K–R)**. Taken from unpublished material from Turégano-López et al. (2021).

This aligns with the findings of Turégano-López et al. (2021), who studied VGAT-positive boutons using FIB/SEM without the use of potassium ferrocyanide. As depicted in Figure 7, the morphology of the synaptic junctions formed by VGAT-positive boutons in this material (without potassium ferrocyanide) is similar to those observed in brain sections using 0.1% potassium ferrocyanide (Figure 4). Therefore, it is recommended to use the latter concentration of potassium ferrocyanide, i.e., 0.1%.

## 4 Discussion

The unambiguous identification of asymmetric (AS) and symmetric (SS) synapses is crucial to unveil the synaptic organization of the brain. It is worth noting that brain tissue fixed with glutaraldehyde allows for the differentiation between AS and SS based on the shape of synaptic vesicles. Specifically, AS typically exhibit round vesicles, whereas some SS display pleomorphic vesicles, including both round and elongated forms (e.g., Peters and Palay, 1996). Nevertheless, we refrain from using glutaraldehyde as the primary fixative due to several drawbacks. Firstly, this fixative is associated with increased background fluorescence and limited antibody penetration into tissues, as demonstrated, for example, by Stradleigh (2015). Additionally, the use of glutaraldehyde can lead to loss of immunogenicity due to the denaturation of certain antigens crucial for our research objectives within the same brain tissue. Furthermore, the high concentrations of glutaraldehyde commonly employed for electron microscopy are incompatible with other methods currently utilized to examine the microanatomy of the brain, such as intracellular injections in fixed tissue. In essence, to optimize the utility of brain tissue in our studies (particularly when dealing with human brain tissue), we prefer to use 4% paraformaldehyde as the primary fixative, as this consistently yields excellent results across various microanatomical methods, including immunocytochemistry and electron microscopy (Domínguez-Álvaro et al., 2018, 2021a; Benavides-Piccione et al., 2020; Montero-Crespo et al., 2021; Benavides-Piccione et al., 2023). Nevertheless, for electron microscopy studies, after the first fixation in paraformaldehyde, sections are postfixed in solutions containing glutaraldehyde.

In the present study, we have described a method to unequivocally identify AS and SS based on morphological criteria of the PSD in brain tissue primary fixed with 4% paraformaldehyde. We have shown that using 0.1% potassium ferrocyanide, the morphology of synaptic contacts can be accurately identified. However, with a higher concentration of potassium ferrocyanide/ferricyanide, some membrane specializations —such as the PSD of SS— become difficult to identify as they may be masked masked by the thicker profiles of non-synaptic membranes. This is probably one of the reasons why many ultrastructural studies that utilized high concentrations of potassium ferrocyanide/ferricyanide (1.5–3.0%) provided no data on SS (e.g., Harris and Stevens, 1989; Hayworth et al., 2014; Yakoubi et al., 2019a; Yin et al., 2020; Gour et al., 2021; Phelps et al., 2021; Peddie et al., 2022; Turner et al., 2022). Several different approaches have been used to overcome the challenge of synapse identification in brain samples with a high concentration of potassium ferrocyanide/ferricyanide. The use of high-resolution transmission electron microscopy in brain samples fixed with a high concentration of glutaraldehyde may facilitate the distinction between PSD of SS and the electron density of non-synaptic membranes (Bromer et al., 2018; Kleinjan et al., 2023). Other studies classify excitatory and inhibitory synapses based on the immunolabeling of the presynaptic neuron (Medalla et al., 2007; Zikopoulos and Barbas, 2007, 2010, 2012; Medalla and Barbas, 2009, 2010, 2014; Collman et al., 2015; Medalla and Luebke, 2015; Wang and Barbas, 2018; Wang et al., 2021; Joyce et al., 2022). Alternatively, several other articles classify synapses as excitatory and inhibitory based on their postsynaptic targets (i.e., dendritic spines and dendritic shafts) (Motta et al., 2019; Karimi et al., 2020; Loomba et al., 2022). A clear preference of glutamatergic axons (forming AS) for dendritic spines and GABAergic axons (forming SS) for dendritic shafts is reported in the literature (reviewed in DeFelipe et al., 2002; for a recent study, see Cano-Astorga et al., 2023). However, this characteristic is often misinterpreted as implying that synapses on dendritic shafts are mostly SS (Motta et al., 2019; Karimi et al., 2020; Loomba et al., 2022). In fact, quantitative analyses of synapses in the neuropil have shown that most synapses on dendritic shafts are AS (∼80%), with relatively few being SS (∼20%) (Beaulieu et al., 1992; Peters et al., 2008; Hsu et al., 2017; Calì et al., 2018; Santuy et al., 2018b; Domínguez-Álvaro et al., 2019, 2021a,b; Yakoubi et al., 2019b; Montero-Crespo et al., 2020, 2021; Cano-Astorga et al., 2021, 2023; Alonso-Nanclares et al., 2023). Therefore, synaptic organization datasets with incorrect assumptions regarding SS identification could introduce an important source of bias.

In conclusion, the use of a lower concentration of potassium ferrocyanide (0.1%), as we propose here, shows an improvement in membrane visualization, while still allowing the PSD of the SS to be identified (Domínguez-Álvaro et al., 2018, 2019, 2021a,b; Montero-Crespo et al., 2020, 2021; Cano-Astorga et al., 2021, 2023). The fact that only VGAT-positive boutons establish SS corroborates the widely accepted correspondence between SS and inhibitory synapses, as well as between AS and excitatory synapses in the cerebral cortex, as reported in previous studies.

## Data availability statement

The original contributions presented in the study are included in the article/Supplementary material, further inquiries can be directed to the corresponding author.

## Ethics statement

The animal study was approved by the European Community Directive 2010/63/EU and the Local Ethics Committee of the Spanish National Research Council (CSIC). The study was conducted in accordance with the local legislation and institutional requirements.

## Author contributions

NC-A: Formal analysis, Investigation, Methodology, Writing – original draft, Writing – review & editing. SP-A: Formal analysis, Investigation, Methodology, Writing – original draft, Writing – review & editing. MT-L: Methodology, Writing – review & editing. JR-R: Methodology, Writing – review & editing. AM-P: Conceptualization, Writing – review & editing. JD: Conceptualization, Funding acquisition, Supervision, Validation, Writing – review & editing.
