# Supplementary material for: Unambiguous identification of asymmetric and symmetric synapses using volume electron microscopy
Source: Front Neuroanat. 2024 Apr 5;18:1348032. doi: 10.3389/fnana.2024.1348032 (PMC11026665; doi:10.3389/fnana.2024.1348032)
Supplement: Supplementary file 1 [file Data_Sheet_1.PDF]

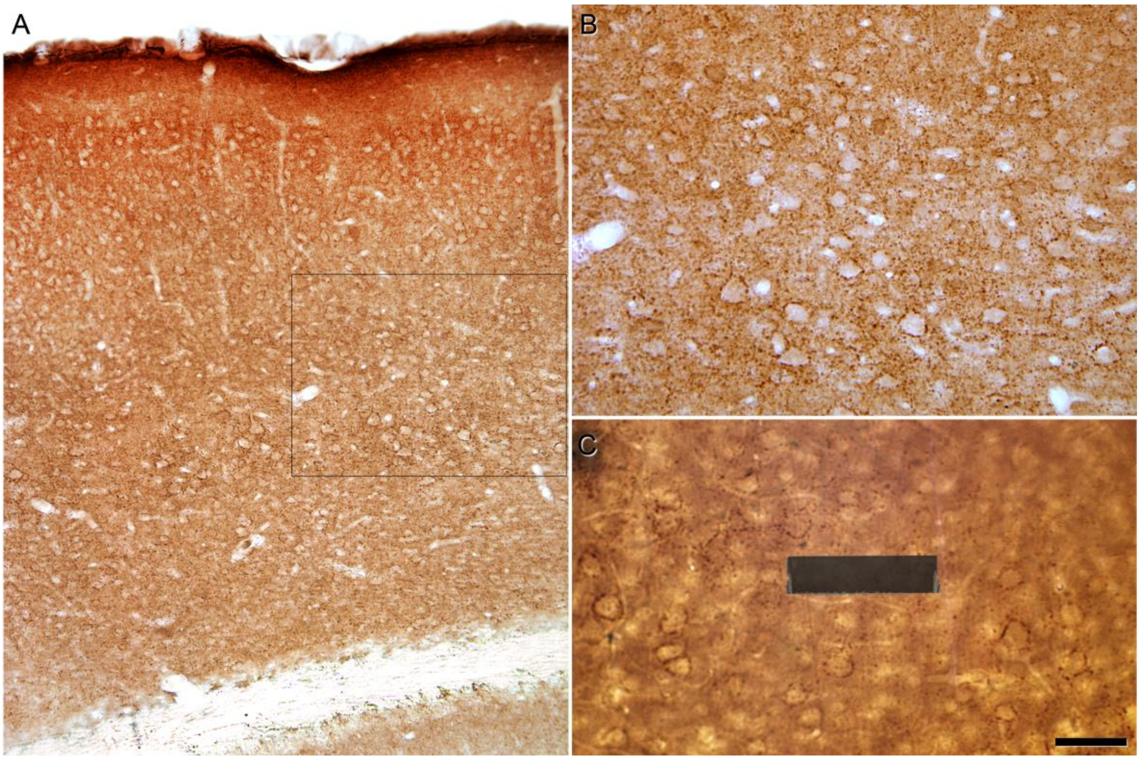

Supplementary Figure 1. Visualization of V-GAT immunoreactivity through light microscopy and selection of the region of interest (ROI). (**A**) Low-power photograph showing the mouse primary somatosensory cortex (S1). Positive V-GAT inmunoreactive puncta can be identified in all layers of the cortex. (**B**) Higher magnification of the boxed area in A, illustrating the distribution of V-GAT puncta in both the neuropil and around soma of unlabeled pyramidal cells. (**C**) Illustration of correlative light-electron microscopy, showing the FIB-SEM sampling ROI, superimposed as a dark trapezoid. Scale bar (in C) indicates 85µm in (A), 42µm in (B) and 50µm in (C).
