# Supplementary material for: Unambiguous identification of asymmetric and symmetric synapses using volume electron microscopy
Source: Front Neuroanat. 2024 Apr 5;18:1348032. doi: 10.3389/fnana.2024.1348032 (PMC11026665; doi:10.3389/fnana.2024.1348032)
Supplement: Supplementary file 2 [file Data_Sheet_2.PDF]

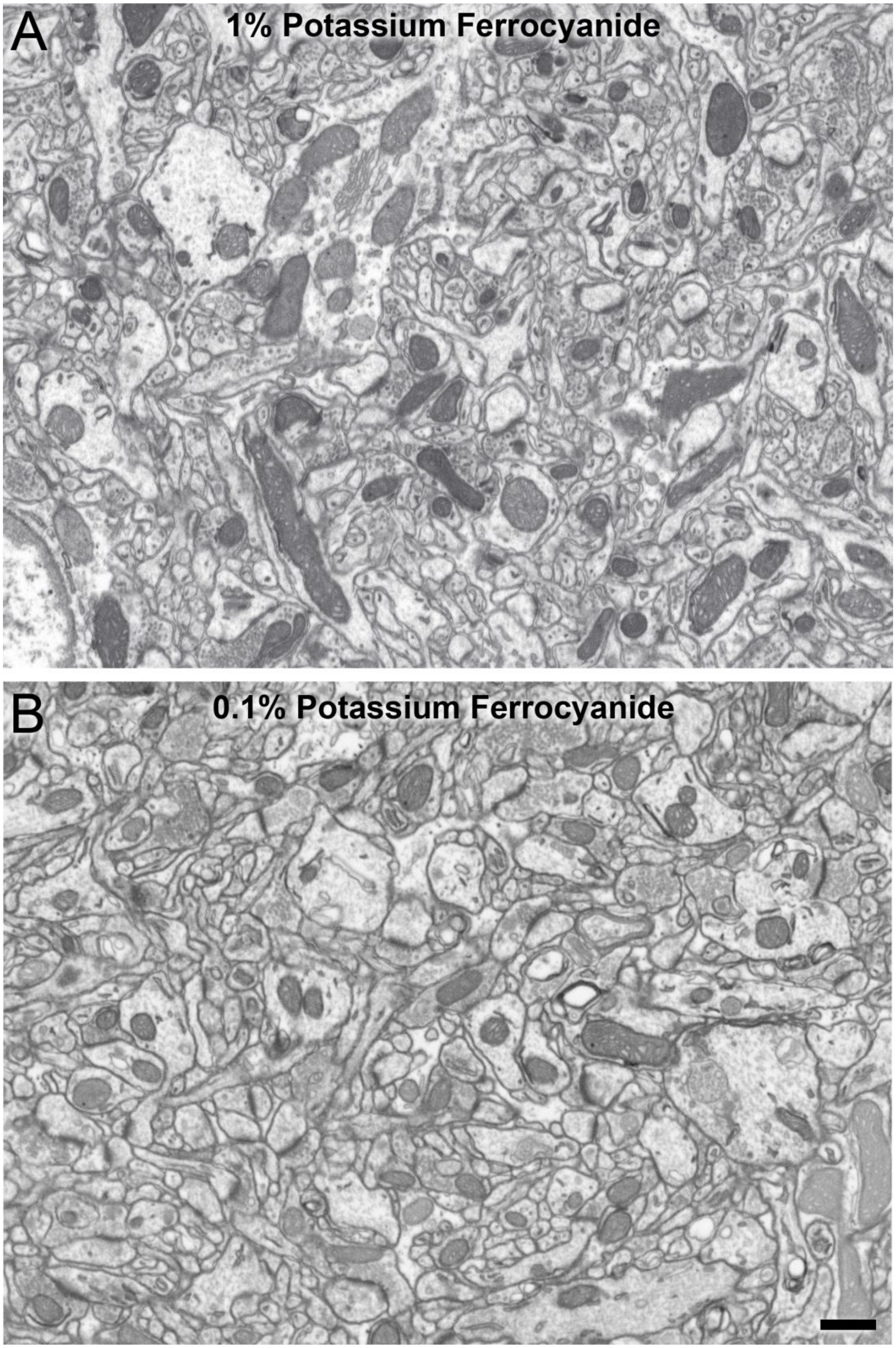

Supplementary Figure 2.1. Ultrastructure of the neuropil from the somatosensory cortex of the mice treated with 1% potassium ferrocyanide (A) or 0.1% potassium ferrocyanide (B). Each image was extracted from the same stack of images as Figure 1 and Figure 2, respectively.

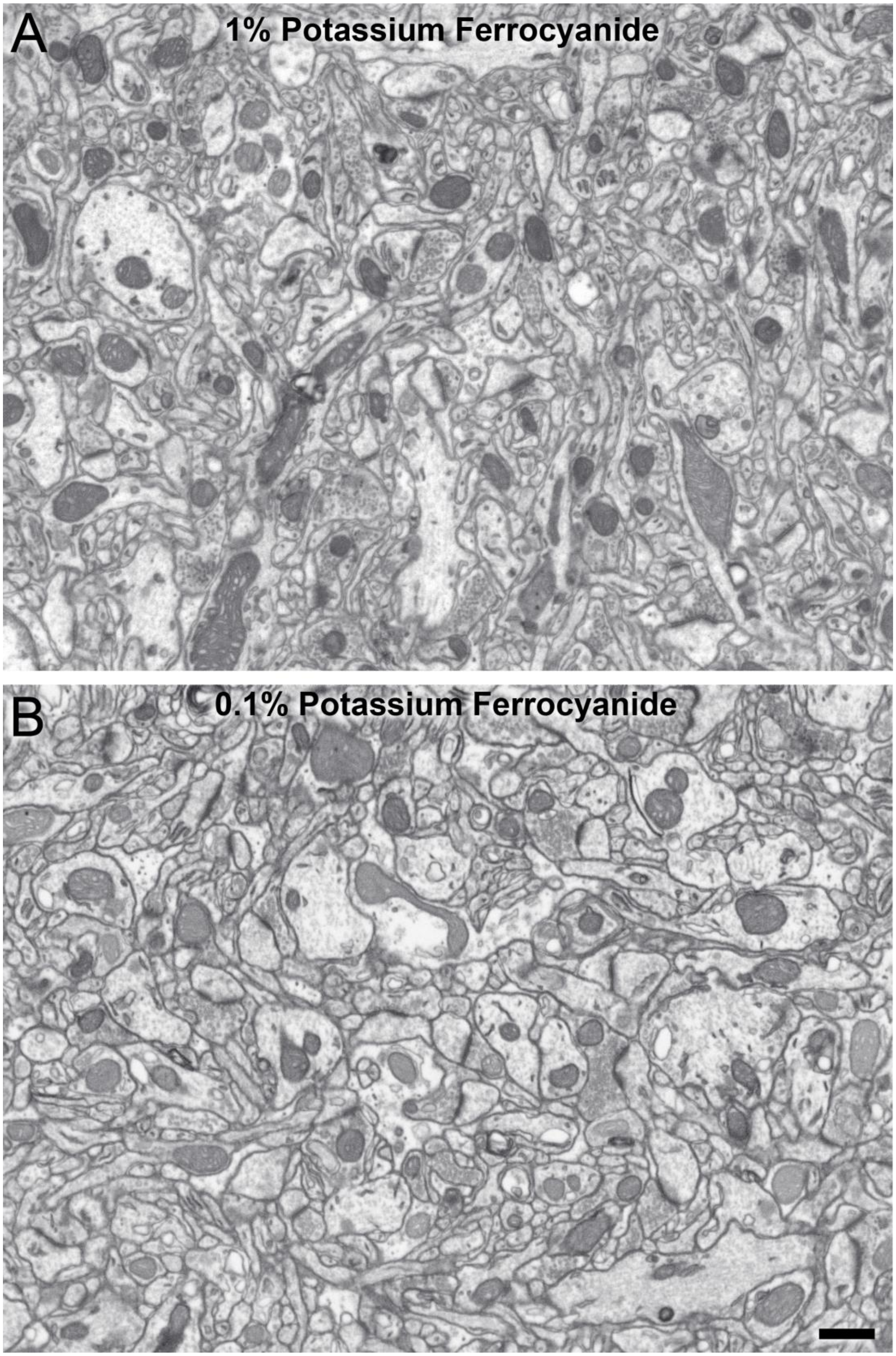

Supplementary Figure 2.2. Ultrastructure of the neuropil from the somatosensory cortex of the mice treated with 1% potassium ferrocyanide (A) or 0.1% potassium ferrocyanide (B). Each image was extracted from the same stack of images as Figure 1 and Figure 2, respectively.

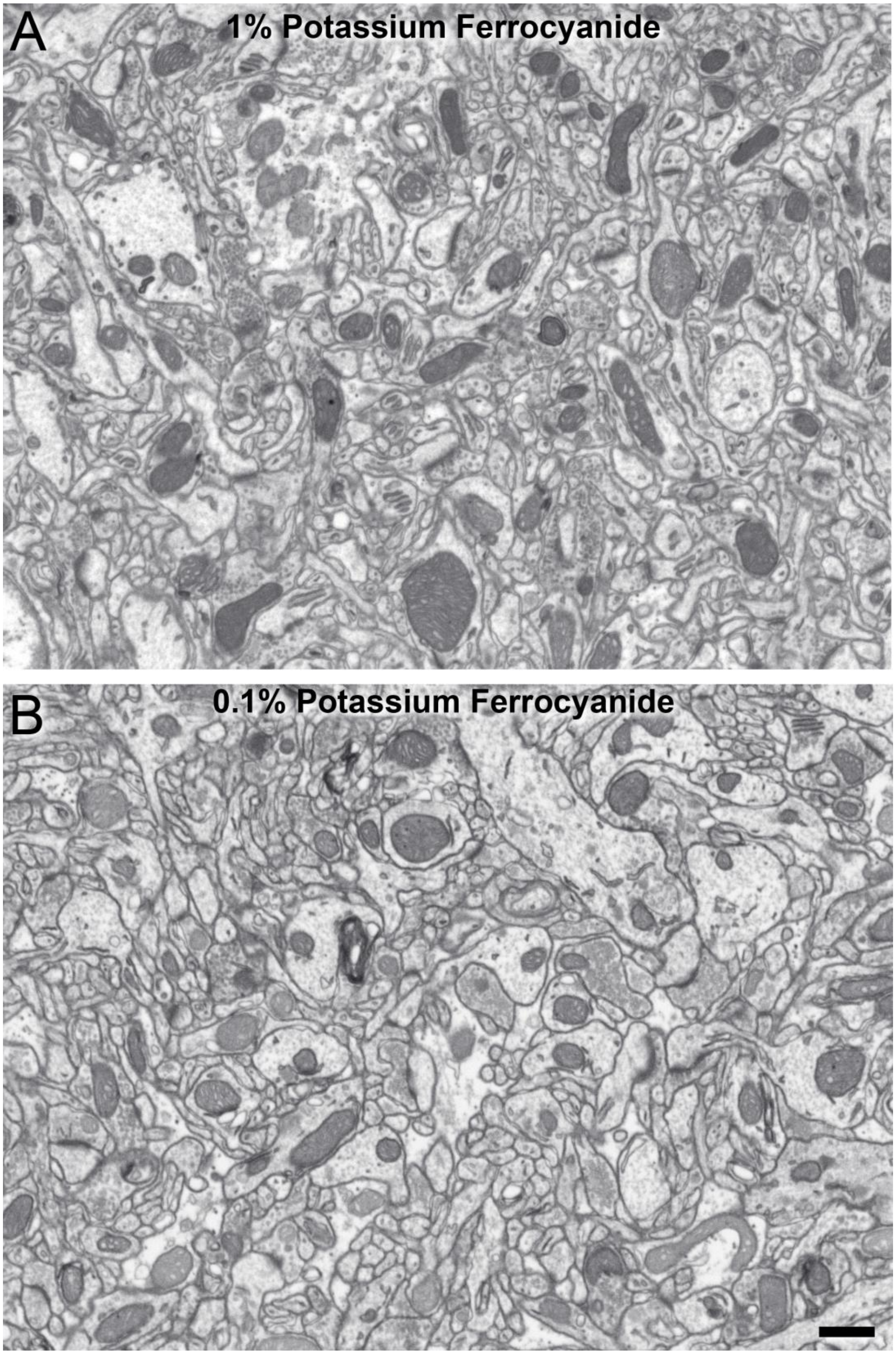

Supplementary Figure 2.3. Ultrastructure of the neuropil from the somatosensory cortex of the mice treated with 1% potassium ferrocyanide (A) or 0.1% potassium ferrocyanide (B). Each image was extracted from the same stack of images as Figure 1 and Figure 2, respectively.

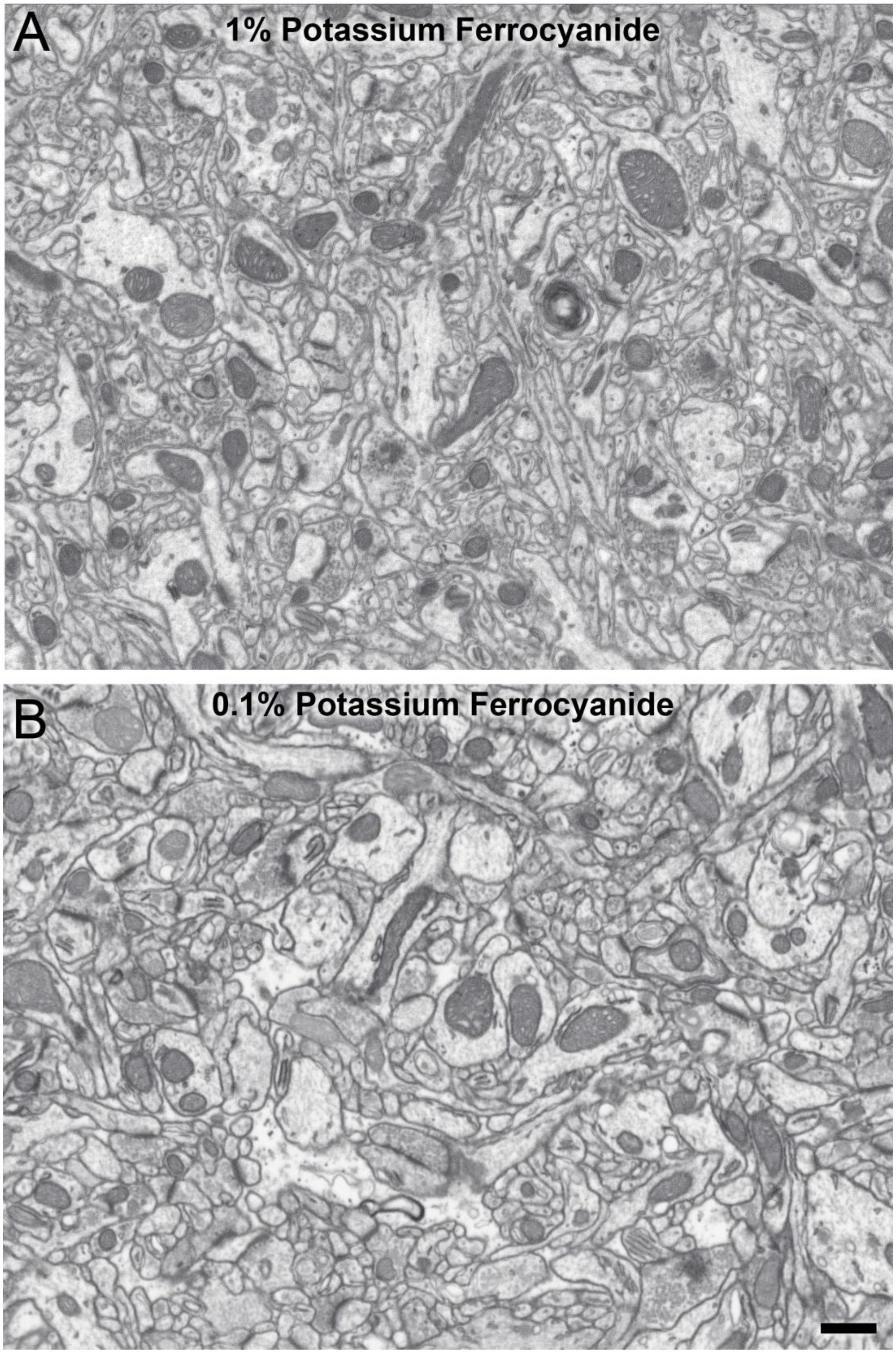

Supplementary Figure 2.4. Ultrastructure of the neuropil from the somatosensory cortex of the mice treated with 1% potassium ferrocyanide (A) or 0.1% potassium ferrocyanide (B). Each image was extracted from the same stack of images as Figure 1 and Figure 2, respectively.
